# Dosimetric comparison of 4 MeV and 6 MeV electron beams for total skin irradiation

**DOI:** 10.1186/1748-717X-9-197

**Published:** 2014-09-06

**Authors:** So-Yeon Park, Beom Seok Ahn, Jong Min Park, Sung-Joon Ye, Il han Kim, Jung-in Kim

**Affiliations:** Department of Radiation Oncology, Seoul National University Hospital, Seoul, Korea; Interdiciplinary Program in Radiation Applied Life Science, Seoul National University College of Medicine, Seoul, Korea; Biomedical Research Institute, Seoul National University Hospital, Seoul, Korea; Institute of Radiation Medicine, Seoul National University Medical Research Center, Seoul, Korea; Center for Convergence Research on Robotics, Advance Institutes of Convergence Technology, Suwon, Korea; Department of Transdisciplinary Studies, Program in Biomedical Radiation Sciences, Seoul National University Graduate School of Convergence Science and Technology, Suwon, Korea; Cancer Research Institute, Seoul National University Hospital, Seoul, Korea; Department of Radiation Oncology, Seoul National University College of Medicine, Seoul, Korea

**Keywords:** Total skin electron irradiation (TSEI), Electron energy, Stanford Technique, Mycosis fungoides

## Abstract

**Background:**

In this study, dosimetric aspects of TSEI consisting of a 4 MeV beam with no spoiler were investigated in comparison to a nominal 6 MeV beam with spoiler, and the potential for clinical applications was evaluated.

**Methods:**

The TSEI technique is based on the Stanford technique, which utilizes a beam configuration of six-dual fields. MOSFETs were used to measure the optimal gantry angle, profile uniformity, and absolute dose at the calibration point. The depth dose curve of the central axis was measured in the treatment plane using EBT2 film. Photon contamination was measured as the dose at 5 cm depth in a solid water phantom relative to the maximum dose using a parallel plate ion chamber. A MOSFET dosimeter placed on the surface of a humanoid phantom, and EBT2 films inserted into a humanoid phantom were used to verify the TSEI commissioning.

**Results:**

Dosimetric aspects of the 4 MeV TSEI beam, such as profile uniformity (±10%) and relative photon contamination (<0.001%), were comparable to those of a 6 MeV TSEI beam. The relative depth dose of the 4 MeV electrons was 81.4% at the surface and 100% at 0.4 cm. For the 6 MeV electrons, the relative depth dose was 93.4% at the surface and 100% from 0.2 cm to 0.4 cm. The calculated B-factor of the 4 MeV TSEI beam was 1.55, and 1.53 for the 6 MeV TSEI. 80% of the prescribed dose was obtained at 0.22 cm depth for the 4 MeV TSEI beam and 0.53 cm for the 6 MeV TSEI beam in the humanoid phantom measurement.

**Conclusions:**

The suggested 4 MeV beam for TSEI could be applied to shallow depth skin diseases and to electron boost as second treatment course.

## Background

Total skin electron irradiation (TSEI) was developed by Stanford University in the 1950s and introduced for the treatment of mycosis fungoides, the most common form of cutaneous T-cell lymphoma which generally affects the skin [1]. Since then, TSEI has been considered to be one of the best treatments for mycosis fungoides and employed for various diseases confined to the skin [2–4]. TSEI has also been extended for the treatment of other cutaneous disease such as Kaposi’s sarcoma and scleromyxoedema [5–9]. The goal is to deliver a relatively uniform dose (e.g., ±10%) to the entire surface of the skin, and not to exceed total photon contamination of 0.7 Gy [10]. In order to deliver the treatment dose at a shallow depth, relatively low energy electrons are considered for TSEI, which is commonly performed with a 6 MeV beam [11–15]. 6 MeV electrons attenuated through a beam spoiler and air are sufficient to deliver the treatment dose to the epidermis and dermis located at a depth of 0.5 cm from the skin surface [16]. For 6 MeV beams the use of a spoiler as a beam scatter-energy degrader is essential to allow adequate skin and depth doses. Increase in surface dose and improved profile uniformity can be obtained by placing the spoiler in front of the patient or mounting it on the gantry head [3, 4, 10, 12, 16]. Proper selection and placement of a spoiler strongly influence the quality of patient treatment, since the spoiler material and position along the beam axis are important in determining the monitor units (MU), profile uniformity, depth dose and relative photon contamination over the treatment plane. The nominal energy for skin electron therapy should range from 4 MeV to 8 MeV [17–20]. Electrons lose approximately 2 MeV/(g/cm2) while passing through the spoiler and air at extended source to surface distance (SSD) so that initially 6 MeV electrons arrive at the patient with an energy of around 4 MeV [21, 22].

The modified Stanford technique has been used in the clinical implementation of TSEI [23]. Our institution implemented TSEI treatment with a six-dual field technique using 6 MeV electrons with an acrylic spoiler of 1 cm and extended SSD of 380 cm.

In this study, TSEI consisting of a 4 MeV beam was investigated with no a spoiler and therefore had an obliquity factor larger than 6 MeV. This obliquity factor is called as B factor which is ratio the treatment skin dose to the calibration point dose for the Stanford technique [24]. The electron energy at the treatment plane was approximately equal to that of an attenuated 6 MeV beam. Furthermore, it reduces the photon contamination. The comprehensive dosimetric aspects for the stated 4 MeV technique were investigated in comparison to the nominal 6 MeV technique, and its potential for clinical application was also investigated.

## Methods

### Geometric Conditions of TSEI

**Figure 1:**
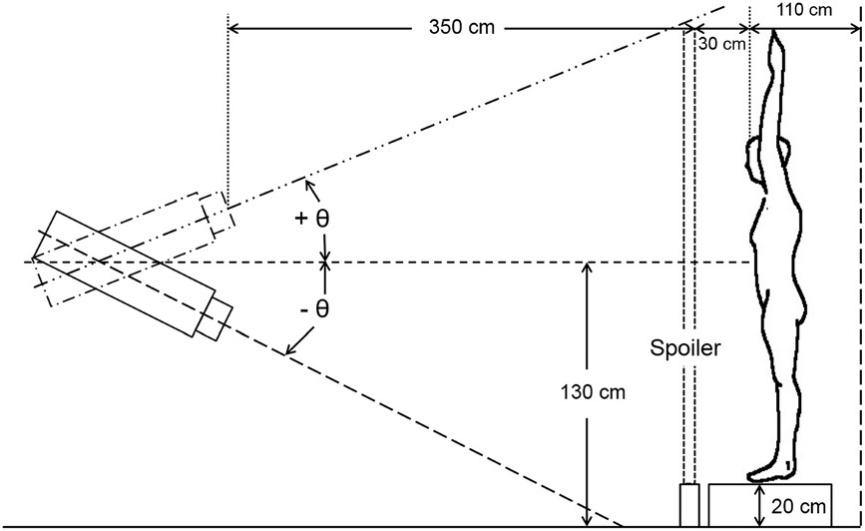
**Positions of treatment apparatus gantry used to generate a dual field for the anterior body position in TSEI.** The spoiler was only used for 6 MeV TSEI beam. The gantry angle (θ) was ± 18.5° and 90 ± 18° for 4 MeV with no spoiler and 6 MeV with spoiler, respectively.

TSEI technique is based on the Stanford technique, which uses a beam configuration of six-dual fields. A Varian Trilogy linear accelerator (Varian Medical Systems, Palo Alto, CA) generated both electron beams (4 and 6 MeV) with field size of 34 × 34 cm^2^, operated under the high dose rate mode (1000 MU/min). In our treatment room, the distance from source to wall was 490 cm from the horizontal beam axis. The position of the spoiler, a home-made acrylic plate (1.05 g/cm^2^) of 106 × 205 × 1 cm^3^, was 30 cm from the treatment plane for 6 MeV, and was not used for 4 MeV. The SSD of 380 cm for TSEI was not influenced by scattering radiation from the walls of the treatment room. With an SSD of 380 cm the dual field was obtained by using two fields for which the gantry is located within ±20° to the 90° gantry angle, which is perpendicular to the treatment plane. Figure 1 presents the patient setup and geometric conditions of the dual field technique. The MOSFET (Best Medical Canada, Ottawa, ON) was cross-calibrated against a NIST-traceable PTW 31013 cylindrical ion chamber (PTW, Freiburg, Germany) in a 9 MeV electron beam, following AAPM TG-51 protocols [25]. The energy dependence of the MOSFET dosimeters was tested before measurement. The MOSFET dosimeters used in this study showed reliable performance in accordance with the results of previous studies [26]. First, the gantry angles which make the most uniform profile of the vertical axis were determined for each TSEI electron beam using the MOSFET measurement with spatial resolution of 0.5° in an acrylic plate located in the treatment plane.

### Dosimetric measurements

Another home-made acrylic plate (1.05 g/cm^2^) of 160 × 205 × 1 cm^3^ which used as total body irradiation (TBI) treatment spoiler was applied as a phantom. The dosimetric measurement was performed by attaching the MOSFET on this phantom. The profile uniformity across and along the patient’s longitudinal axis, with optimized gantry angles for each electron beam, were investigated with spatial resolutions of 2 cm and 5 cm for a central area of 40 cm, and outside of a central area using the MOSFET measurements, respectively. The absolute dose at the calibration point, which was located in treatment plane, was also defined with MOSFET measurements.

The percentage depth dose (PDD) curve in Solid Water™ (GAMMEX RMI, Middleton, WI) of the central axis for the 4 MeV and 6 MeV TSEI beams was measured for dual fields in the treatment plane using radiochromic EBT2 film (ISP, Wayne, New Jersy, USA). The film was inserted in the center of solid water phantom of 10 cm parallel to the 90° gantry angle. Photon contamination for 4 MeV with no the spoiler, and 6 MeV with spoiler was measured as the dose at 5 cm depth in a solid water phantom relative to the maximum dose using the Advanced Markus parallel plate ion chamber, type M34045 (PTW, Freiburg, Germany). In order to prevent the cable-induced effects on ion chamber measurements in large electron fields, known as the “Cable effect”, lead shielding of 0.5 cm thickness was applied to the chamber cable [27].

### Dosimetric commissioning and QA

Finally, with patient setup and geometric conditions of the six dual-field technique, it is essential to determine the multiplication factor (*B*-factor) for the delivered dose at the patient surface. This factor, which relates the treatment skin dose to the calibration point dose, was calculated from measurements from a MOSFET placed on the surface of a humanoid phantom (CIRS Inc., Norfolk, VA). Phantom setup was made identical to the treatment setup by changing the positions every 60° about the patient’s longitudinal axis. Eight MOSFETs were attached on the phantom’s surface; at each of four locations (anterior, posterior, left and right lateral) in both the chest section and the abdominal section.

Three EBT2 films were inserted in the head, chest, and abdominal sections of the humanoid phantom to verify the TSEI commissioning. The film was then scanned using an Epson 1000X flatbed scanner (Epson America, Inc. Long Beach, CA) and in-house software was used to assess the depth dose distribution and isodose curves within the phantom.

## Results

### Profile uniformity

**Figure 2:**
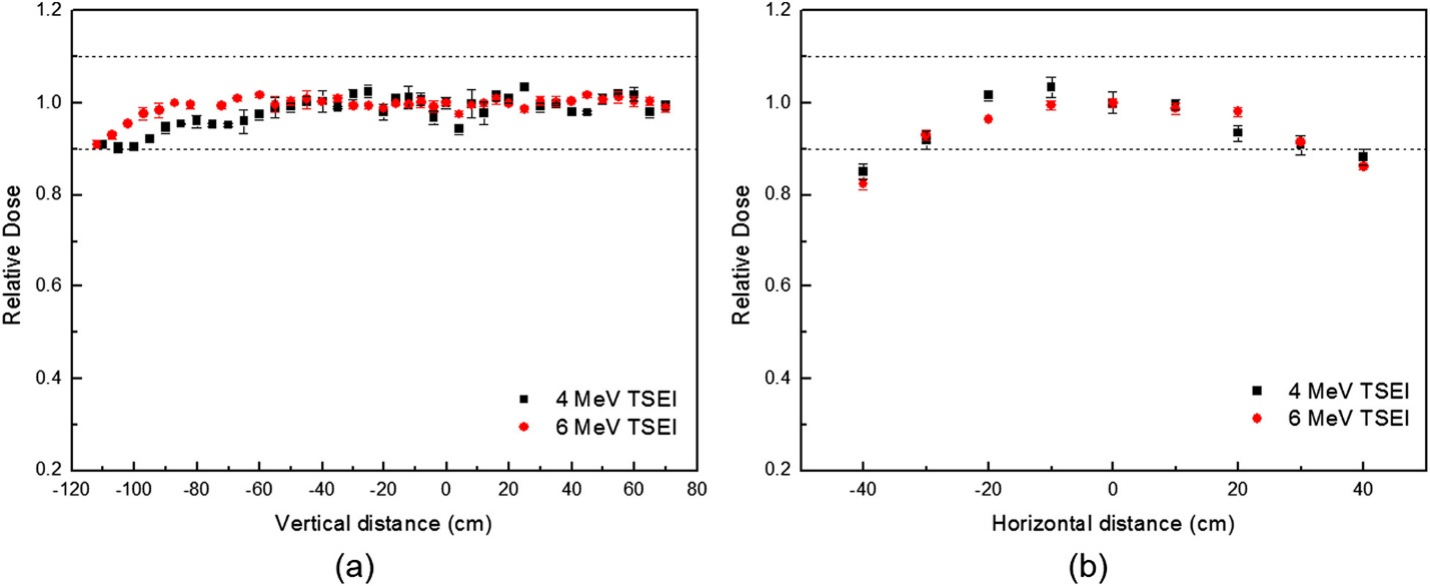
**Uniform vertical and horizontal profiles for TSEI beams.** Profiles of TSEI treatment of vertical direction **(a)** and horizontal direction **(b)** are shown.

The optimized treatment fields were at 90 ± 18.5° and 90 ± 18° for 4 MeV with no spoiler and 6 MeV with spoiler, respectively. With these dual fields, the results of profile uniformity are shown in Figure 2. In the vertical (inferior to superior) profile, uniformity of ±10% was demonstrated for 4 MeV and 6 MeV TSEI beams up to the end of the spoiler from 20 cm above the floor. Thus, a wooden patient support device of 20 cm height was designed to reduce scatter from the floor, and to indicate the patient’s treatment positions. The profile variations of 4 MeV with no a spoiler are much greater than that of 6 MeV with a spoiler. In the horizontal (left to right) profile, uniformity of ±10% was demonstrated for 4 MeV and 6 MeV TSEI beams within 70 cm width. The profiles for both beams were similar and shaped like a parabola.

### PDD and Photon contamination

**Figure 3:**
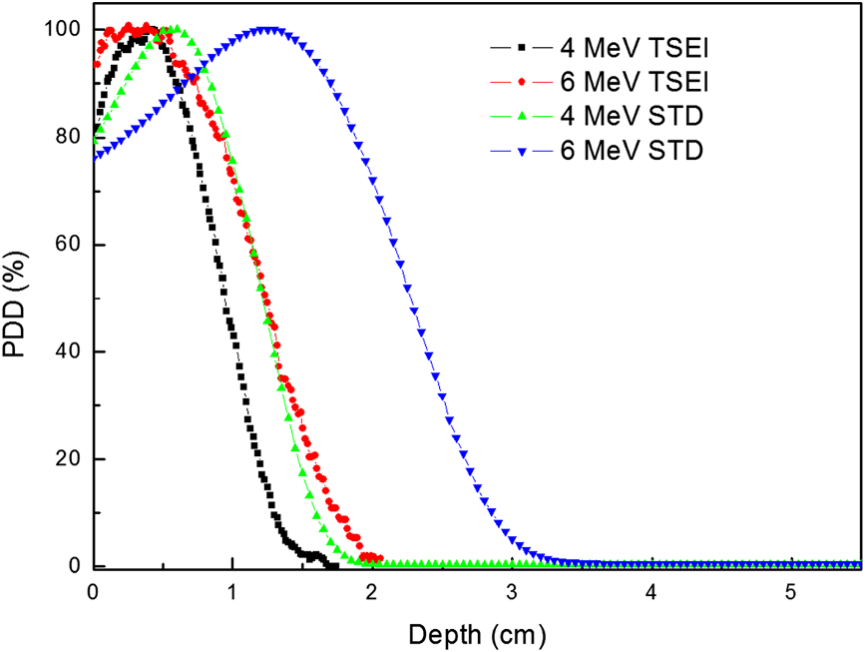
**Relative depth dose curve for central axis with dual-fields.** The STD (standard) beam was from the 100 cm SSD and 10 cone field.

With TSEI treatment fields, the relative depth dose of 4 MeV electrons was 81.4% at the surface and 100% at 0.4 cm. For 6 MeV electrons, the relative depth dose was 93.4% at the surface and 100% from 0.2 cm to 0.4 cm. The depth of maximum dose was identical for both TSEI beams since the maximum energy of 6 MeV electrons attenuated by the 1 cm spoiler was 4 MeV. The PDD results are shown in Figure 3. The 4 MeV electrons with no the spoiler had a maximum penetration depth 1.3 cm. For 6 MeV with the spoiler, the maximum penetration was 1.9 cm. The mean energy  was calculated from R_50_ according to AAPM TG-70 [28]. The mean energy was 2.9 MeV and 3.2 MeV for 4 MeV and 6 MeV TSEI beams, respectively. For photon contamination, the ion chamber detected a signal which was contaminated with some level of noise (<0.001%). Table 1 is a tabulation of the treatment beam parameters.

**Table 1:** **Treatment beam quality parameters**

Electron beam	R_50_(cm)	D_max_(cm)	(MeV)	R_p_(cm)	Contamination (%)
Standard 4 MeV	1.2	0.6	3.2	1.6	0.3
Standard 6 MeV	2.3	1.3	5.5	2.9	0.6
4 MeV TSEI	0.95	0.4	2.9	1.3	< 0.001
6 MeV TSEI	1.2	0.4	3.2	1.6	< 0.001

Calculated using TG-70: .

### Phantom measurements and verification

**Figure 4:**
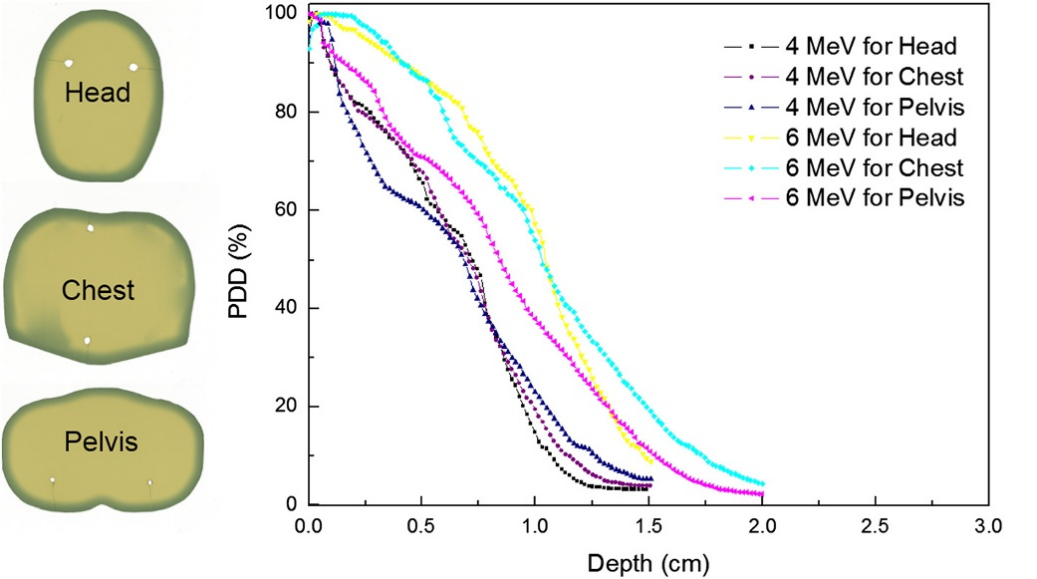
**Relative depth dose curve for six-dual fields using film within three section of humanoid phantom.**

During one cycle (two treatment days) of TSEI, MOSFET detectors placed on the surface of the humanoid phantom were irradiated based on calculated MUs at the calibration point using the six dual-field technique. The average measured doses at the eight points were normalized to the prescription dose (200 cGy). Table 2 summarizes the resulting dose verification for the 4 MeV TSEI beam. The dose difference was calculated between the normalized dose and prescription dose. The maximum dose discrepancy of in-vivo dosimetry was 8.5% higher than the prescription dose and within the acceptable tolerance level of 10%. The calculated B-factor of the 4 MeV TSEI beam was 1.55. Table 3 summarizes the resulting dose verification for the 6 MeV TSEI beam. The maximum dose discrepancy was 6.5% lower than the prescription dose. The calculated B-factor of the 6 MeV TSEI beam was 1.53. The B-factors were comparable for both TSEI beams in this study. Using the B-factor, the treatment MUs were finally determined for both TSEI beams. The films inserted in the humanoid phantom were used for relative dose measurements. The periphery of the film was uniformly irradiated from the TSEI beams. PDDs from the irradiated films are shown in Figure 4. The maximum dose occurred at the surface of the phantom. At all three parts of the phantom, PDDs of the 4 MeV TSEI beam were steeper than that of the 6 MeV TSEI beam. The depth at which 80% of the prescribed dose was 0.22 cm for 4 MeV TSEI and average 0.53 cm for 6 MeV TSEI, which was the average depth of three films. The maximum penetration depth of the TSEI beams were 1.5 cm and 2 cm for 4 MeV and 6 MeV TSEI beams, respectively.

**Table 2:** **MOSFET dose measurement 4 MeV TSEI for dosimetric commissioning and treatment MUs**

Detector location	1^st^day dose (cGy)	2^nd^day dose (cGy)	Total dose (cGy)	Normalized dose (cGy)	Dose difference (%)
ANT of Chest	110	201	311	200.8	0.4
Right of Chest	140	168	308	198.9	-0.6
POST of Chest	197	103	300	193.7	-3.1
Light of Chest	135	160	295	190.5	-4.8
ANT of Umbilicus	115	221	336	216.9	8.5
Right of Umbilicus	154	161	315	203.4	1.7
POST of Umbilicus	203	104	307	198.2	-0.9
Light of Umbilicus	151	155	306	197.6	-1.2
Average			309.8	200.0	0.0

First day beams: AP, LPO, and RPO Second day beams: PA, LAO, and RAO.

**Table 3:** **MOSFET dose measurement 6 MeV TSEI for dosimetric commissioning and treatment MUs**

Detector location	1^st^day dose (cGy)	2^nd^day dose (cGy)	Total dose (cGy)	Normalized dose (cGy)	Dose difference (%)
ANT of Chest	112	106	218	200.7	0.4
Right of Chest	114	194	307	198.0	-1.0
POST of Chest	138	165	303	189.3	-5.4
Light of Chest	185	105	290	202.8	1.4
ANT of Umbilicus	163	147	311	199.4	-0.3
Right of Umbilicus	111	195	305	211.7	5.9
POST of Umbilicus	161	163	324	186.3	-6.8
Light of Umbilicus	178	107	285	211.7	5.9
Average			306.2	200.0	0.0

First day beams : AP, LPO, and RPO Second day beams: PA, LAO, and RAO.

## Discussion

The 4 MeV electron beam with no a spoiler was investigated in implementing a clinical TSEI in this study. The electron measurement at extended SSD has a larger uncertainty and more setup variation than the electron measurement with nominal SSD. Thus, all measurements were performed three times and the average of the values was applied in this study.

The comprehensive dosimetric aspects for the 4 MeV TSEI beam were compared to a nominal 6 MeV TSEI technique. The dosimetric aspects of the 4 MeV TSEI beam, such as profile uniformity and relative photon contamination, were comparable to those of the 6 MeV TSEI beam, which could be implemented a clinical treatment. In the PDD, however, 80% of the prescribed dose was obtained at 0.22 cm depth in humanoid phantom measurements, which was shallower than the epidermis and dermis locations of 0.5 cm. It is limited to use for large tumor in T3 stage of CTCL TNM classification.

The required penetration depth is usually thought to vary with the stage and type of disease and may vary over the body [29]. It also depends on the part of the body, the epidermis is thinnest on the eyelids at 0.5 cm, and thickest on the palms and soles at 0.15 cm, and dermis is 0.03 cm on the eyelid and 0.3 cm on the back. The 4 MeV TSEI beam could be used for skin diseases located in shallow depths of less than 0.3 cm. Moreover, an electron boost was concurrently administered to under dosage regions such as the top of the patient’s head and perineum. The 4 MeV TSEI beam, therefore, could be also used for the second treatment course based on region of interest. On the other hand, increase of total MU causes treatment time to rise and longer treatment times may give rise to instability of patient posture.

## Conclusions

The suggested 4 MeV beam for TSEI could be applied to shallow depth skin diseases and to electron boost as second treatment course.

## Abbreviations

TSEI: Total skin electron irradiation
MU: Monitor unit
SSD: Source to surface distance
PDD: Percentage depth dose
TBI: Total body irradiation.
